# Leptin accelerates BMSC transformation into vertebral epiphyseal plate chondrocytes by activating SENP1‐mediated deSUMOylation of SIRT3

**DOI:** 10.1002/2211-5463.13539

**Published:** 2023-01-13

**Authors:** Xiaomiao Li, Xiaodong Fu, Hao Li, Yingjian Gao, Weili Wang, Zude Liu, Yi Shen

**Affiliations:** ^1^ Department of Orthopedics, Renji Hospital, School of Medicine Shanghai Jiaotong University China

**Keywords:** BMSCs, chondrocytes, hypoxia condition, leptin, SENP1, Sirutin3

## Abstract

Bone marrow mesenchymal stem cells (BMSCs) are capable of multidirectional differentiation, and engrafted BMSCs can be used to replace damaged chondrocytes for treatment of intervertebral disc disease. However, chondroblast differentiation of implanted BMSCs is inhibited by the anoxic environment of the articular cavity. Here, we found that leptin enhanced the transformation of BMSCs into chondrocytes under hypoxic conditions. BMSCs isolated from mice were cultured in medium supplemented with leptin under hypoxia. The expression of MFN1/2 and OPA1 were increased only in BMSCs cultured in an anoxic environment. In addition, in hypoxic environments cell energy metabolism relies on glycolysis regulated by leptin, rather than by mitochondrial oxidation. The expression of the de‐SUMOylation protease SENP1 was elevated, leading to SIRT3‐mediated activation of PGC‐1α; these processes were regulated by CREB phosphorylation, and promoted mitochondrial fusion and cell differentiation. The chondrogenic activity of BMSCs isolated from *SIRT3*‐knockout mice was lower than that of BMSCs isolated from wildtype mice. Implantation of *SIRT3*‐knockout murine‐derived BMSCs did not significantly improve the articular cartilage layer of the disc. In conclusion, the hypoxic microenvironment promoted BMSC differentiation into chondrocytes, whereas osteoblast differentiation was inhibited. SENP1 activated SIRT3 through the deSUMOylation of mitochondria and eliminated the antagonistic effect of SIRT3 acetylation on phosphorylation. When phosphorylation activity of CREB was increased, phosphorylated CREB is then transferred to the nucleus, affecting PGC‐1α. This promotes mitochondrial fusion and differentiation of BMSCs. Leptin not only maintains chondrogenic differentiation homeostasis of BMSCs, but also provides energy for differentiation of BMSCs under hypoxic conditions through glycolysis.

## Graphical abstract

Leptin mediates the transformation of BMSCs into chondrocytes under hypoxia. This process depends on SENP1 to remove SUMO of SIRT3 and activate SIRT3 deacetylase. Activated SIRT3 promotes translocation of phosphorylated CREB into the nucleus and indirectly initiates PGC‐1α.

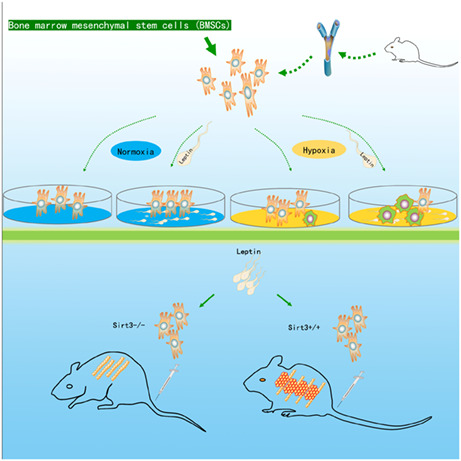

**Abbreviations**
BMSCs: bone marrow mesenchymal stem cells
ECAR: rate of extracellular acidification
FCCP: carbonylcyanide‐4 (trifluoromethoxy)phenylhydrazone
IVDD: intervertebral disc degeneration
LEP: leptin gene
OCR: oxygen consumption rate
PGC‐1α: preceptor (PPAR) γ coactivator 1α
SENP1: De‐SUMOylation protease SUMO‐specific protease1
SIRT3: Sirutin3
TEM: transmission electron microscopy

In line with the worldwide aging trend, diseases associated with low back pain are growing. Intervertebral disc degeneration (IVDD) is often one of the main causes of low back pain [1, 2]. The degeneration of intervertebral disc cartilage is one of the most critical factors that causes IVDD [3]. Therefore, to find an alternative to repairing the degenerating chondrocyte layer of the disc has become a useful treatment for low back pain. In bone marrow stem cells (BMSCs), the inherent properties include chondrogenic transformation potential, deemed as potential vectors of a modifying treatment in degenerative disc disease [4, 5]. Therefore, it is possible to use BMSCs as an alternative source of degenerative chondrocytes in replenishing degenerative disc degeneration [6, 7]. However, the low survival rate and multiple differentiation of BMSCs in the hypoxic environment became the greatest obstacle to therapy [8, 9, 10]. How to improve the survival rate of transplanted BMSCs and the orientation of chondroblast differentiation became particularly important in transplantation therapy.

Leptin is a 16 kDa adipokine, and is encoded by the leptin gene (*LEP*) [11, 12]. The leptin receptor (also known as OB‐R) contains a transmembrane domain, belongs to the family of cytokine‐like receptors, a single transmembrane domain [13]. Leptin exert their biological effects by binding to the specific leptin receptor (Ob‐R), regulates the signal pathways involved in cell functioning, differentiation, and proliferation [14, 15, 16]. Leptin receptors are also found to be expressed on the surface of bone marrow stem cells (BMSCs) [17]. Previous reports show that leptin promotes cellular glycolysis to provide the energy necessary for cellular activities under hypoxic conditions [18, 19]. In addition, glycolysis is widely considered as a pro‐differentiation metabolic pathway [20]. Therefore, we speculated that leptin was an effective source of regulating BMSCs energy metabolism and affecting the differentiation of BMSCs cells under the hypoxia environment.

Mitochondrial biogenesis plays an important role in the mitochondrial dynamics required during cell differentiation [21]. The peroxisome proliferator‐activated receptor gamma coactivator‐1α (PGC‐1α), a nuclear receptor coactivator, stimulate the mitochondrial biogenesis, and play multiple roles in diverse metabolic events [22, 23]. PGC‐1α, acts as a transcriptional co‐activator, and plays an important role in mitochondrial fusion [24]. In addition to transcriptional activation by CREB [25], it has been shown that SIRT1 (SIRT1) also increases PGC‐1α expression and activates it by direct phosphorylation [26]. SIRTuins are protein deacylases including the SIRT family (SIRT1‐7) [27]. Mitochondrial energy metabolism is regulated by SIRT1 as well as SIRT3 [28]. SIRT1 promotes the deacetylation of PGC‐1α, which is an activated state of PGC‐1α during mitochondrial biogenesis [29]. SIRT3, a main mitochondrial acetyllysine deacetylase, is also a SUMOylated protein in mitochondria in physiological states [30]; it has wide‐ranging roles in cell metabolism and differentiation [31]. SIRT3 acts directly as an activator of essential proteins for oxidative phosphorylation and indirectly as an activator of PGC‐1α [31].

In the present study, we demonstrated that leptin stimulated an increase in the BMSCs chondrocyte markers, SOX9 and collagen, under hypoxia and chondrogenic differentiation. It also promotes glucose absorption, increases glycolysis and regulates cellular energy metabolism. However, interestingly, the cellular pathway affected by SIRT1 did not change significantly. The upregulated SNEP1 stimulated by the anoxic microenvironment promoted the energy metabolism required for mitochondrial fusion, renewal, and differentiation through the SIRT3/CREB/PGC‐1α axis and accelerated the progress of cell differentiation. This pathway involves increasing SNEP1 protein expression, while SENP1 regulates SIRT3 by deSUMO and activates SIRT3 deacetylation. SIRT3 also stimulates CREB deacetylases and promotes CREB Phosphorylation activity, and then phosphorylated CREB transferred into the nucleus to activate the PGC‐1α promoter.

## Materials and methods

### Animal studies

All animal experiments were approved by the Animal Ethics Committee of Shanghai Jiaotong University School of Medicine (ethics approval number: WYLS2022‐20). Eight‐week‐old male C57BL6/J mice were used for these studies. Mice were housed in well‐ventilated cages under a 12‐h light–dark cycle at 24 ± 2 °C with free access to food and water. Three to five mice were raised in groups in each cage, which were obtained from the Zhejiang Medical Animal Centre (Hangzhou, China), weighing ~20 g. To develop the injury‐induced disc degeneration models, all the mice used for modeling were preanesthetized with isoflurane (2% induction and 1.5% maintenance) and lidocaine (5 mg·mL^−1^) was injected subcutaneously before surgery, and then the tail disc Co6/7 was exposed by making an approximately 1‐inch longitudinal incision along the outer side of the tail. Next we inserted a needle (Model Number: 16G) into the disc for 1.2 mm and remained in the disc for about 30 s before exiting. After this procedure, the muscle and skin were sutured and antibiotic ointment was applied to the wound margin. A homoeothermic blanket system was used during the whole procedure to maintain the core body temperature at approximately 37 °C until the mice woke up. The mice were given ticarcillin after surgery (50 mg·kg^−1^, IP, for 2 days) as postoperative care.

### Primary cells isolation, culture, and glucose/hypoxia injury

Mice BMSCs were isolated and harvested from C57BL/6 mice. The ends of bones were cut and the marrow was flushed from femurs and tibiae. BMSCs were cultured in Dulbecco's Modified Eagles Medium (DMEM) with 10% fetal bovine serum (FBS) and 1% penicillin–streptomycin at 37 °C with 5% CO_2_, then BMSCs were isolated with the adherence separation method. BMSCs were treated with glucose‐free DMEM medium and leptin/normoxia, no‐leptin/normoxia, leptin/hypoxia, or no‐leptin/hypoxia different culture conditions for 48 h to simulate lack of oxygen and glucose environment. The leptin concentration in the medium was set as 100 ng·mL^−1^ at 37 °C for 24 h and 50 ng·mL^−1^ under the hypoxia condition for 48 h; the hypoxia condition was 0.5% O_2_/5% CO_2_.

### Animal treatment

On the day before surgery, all mice were fasted for 24 h. On the surgery day, all the model mice were preanesthetized with isoflurane (2% induction and 1.5% maintenance), except for sterile normal saline‐treated mice used as a normal control group, The treatment group mice were implanted into about 30 μL cell suspensions containing BMSCs 1 × 10^6^ (with 10 ng·mL^−1^ leptin) through a 1‐mL sterile syringe with a 16G needle. BMSCs were slowly injected into the joint cavity of the mice tail disc Co6/7. After withdrawal of needles, tissue adhesives were used to block puncture holes to prevent cell outflow. The mice were sutured muscle and skin tissue to continue feeding after wound disinfection. After the operation, ticarcillin was injected to prevent infection.

### Metabolic flux analyses using seahorse XFe96 analyzer

Mitochondrial rate of extracellular acidification (ECAR) and oxygen consumption rate (OCR) were measured with the XFe96 Flux Analyzer (Agilent Technologies, Santa Clara, CA, USA). BMSCs were plated in the Matrigel precoated 96‐well plate at a density of 100,000 cells per well 24 h prior. Before putting it on the machine, media were replaced with 175 μL fresh DMEM medium. Seahorse analyzer injection ports were filled with 1 μm oligomycin, 1 μm carbonylcyanide‐4 (trifluoromethoxy) phenylhydrazone (FCCP), or 0.5 μm each of rotenone and antimycin A for OCR. First, oligomycin was added into the wells for adenosine‐5′‐triphosphate (ATP) synthase inhibition; FCCP acts as an uncoupling agent to prevent ATP synthesis and OCR will arrive at its maximum level. The FCCP‐stimulated OCR can be used to calculate spare respiratory capacity. Finally, rotenone and antimycin A inhibit complex I and III, respectively, of the electron transport chain. Nonmitochondrial respiration can be calculated.

The extracellular acid produced by cells was measured through glycolysis and tricarboxylic acid cycle (ECAR). First, measured basal acidification, contributed to by pyruvic acid and carbonic acid at the start of the assay. Once added, 10 mm glucose glycolysis is initiated. Subsequently, the pyruvic acid and lactic acid production increase, resulting in acidification. Next, we added 1 μm oligomycin to block ATP production from mitochondrial respiration. This process allowed the glycolytic capacity to be established. Lastly, we added 50 mm 2‐deoxy‐glucose (2‐DG) as a glycolysis inhibitor for the measurement. Glycolytic capacity was calculated by the difference between ECAR after the injection of 1 μm oligomycin, and the basal ECAR.

### Flow cytometry analysis of the BMSCs

BMSCs were identified by cell surface markers. Briefly, BMSCs were digested with 0.25% trypsin to a concentration of 1 × 10^6^ cells·mL^−1^, which was rinsed and resuspended in cold phosphate‐buffered saline (PBS). We added fluorescently‐labeled antibodies: CD34 (BD, Franklin Lakes, NJ, USA), CD45 (Biolegend, San Diego, CA, USA) into cell suspensions for 30 min at 4 °C protected from light. Afterwards, the BMSCs were washed twice with cold PBS and assessed via a flow cytometry (Becton Dickinson, Franklin Lakes, NJ, USA).

### Immunostaining

Paraffin sections (5 μm thickness) were used for immunostaining. Briefly, sections were deparaffined in xylene for 10 min and then were dewaxed in fresh xylene for 10 min, hydrated in ethanol from 95% to 75% ethanol, and washed with distilled water. Then antigen was repaired with the microwave heating method (heating in 0.01 m citrate buffer/pH 6), and incubated with blocking buffer (10% normal goat serum, 5% bovine serum albumin [BSA], and 0.1 Triton X‐100 in PBS) for 1 h at room temperature. The slides were incubated with primary antibodies overnight at 4 °C, followed by incubation with Alexa 488 fluorophore or HRP‐Rabbit/Mouse secondary antibodies (Jackson Immuno Research Laboratories, West Grove, PA, USA). To quantify the number of positive cells, they were counted from on six different fields per experimental group under the microscope on well‐oriented sections.

### Quantitative real‐time polymerase chain reaction (qPCR)

Total RNA was extracted using Trizol reagent (Invitrogen, Carlsbad, CA, USA, Thermo Fisher Scientific, Wilmington, DE, USA) and the RNA concentration quality were detected by a Nanodrop spectrophotometer (Thermo). The reverse‐transcribed cDNA samples were diluted 10‐fold, forward and reverse primers corresponding to different individual were added into the qPCR reaction system. Relative gene expression levels were measured by 2× SYBR Green qPCR Master Mix (Takara, Dalian, China), the data were analyzed using the $2^{-\Delta \Delta C_{T}}$ method.

### Western blot

We collected the cells or tissues from each group, washed in cold PBS twice, tissue or cell total protein extracts were obtained by ripa lysis buffer (with protease and phosphatase inhibitors), the total protein concentration was calculated by BCA assay. All the proteins were firstly separated by SDS/PAGE gel electrophoresis according to different molecular weight, and then the proteins in the gel was transferred to PVDF membrane, PVDF membranes were incubated with primary and secondary antibodies sequentially. After washing with TBST for 3 times, the protein bands were exposed by chemiluminescence method with enhanced chemiluminescence (Millipore, Billerica, MA, USA), the gray levels of protein bands were analyzed using image j (NIH, Bethesda, MD, USA) software.

### TEM

Transmission electron microscopy (TEM) was used to detect the BMSCs mitochondrial network ultrastructure. Specimens were fixed with 2.5% glutaraldehyde at 4 °C for > 4 h, and then exposure to 1% OsO4 for 1–2 h. Next, the specimens were dehydrated by ethanol from 50% to 100%, acetone infiltration overnight. After infiltration, the specimens were embedded in resin and sectioned with a Leica EMUC7 (Leica, Wetzlar, Germany). At last, the sections were stained with uranyl acetate and alkaline lead citrate, the image procured at ×30,000 magnification by TEM (Hitachi Model H‐7650, Hitachi, Tokyo, Japan). The mitochondrial length and fusion degree were measured.

### Statistical analysis

All data are presented as mean ± SEM. Data analysis and statistical analysis were performed by graphpad prism (version 8.0; San Diego, CA, USA). Student's *t*‐test was used for comparison of two datasets and one‐way ANOVA for three or more sets of data. *P* < 0.05 was defined as statistically significant. All the experiments were run at least three times.

## Results

### Leptin promoted hypoxia‐conditioned BMSCs transformation *in vitro*

**Fig. 1:**
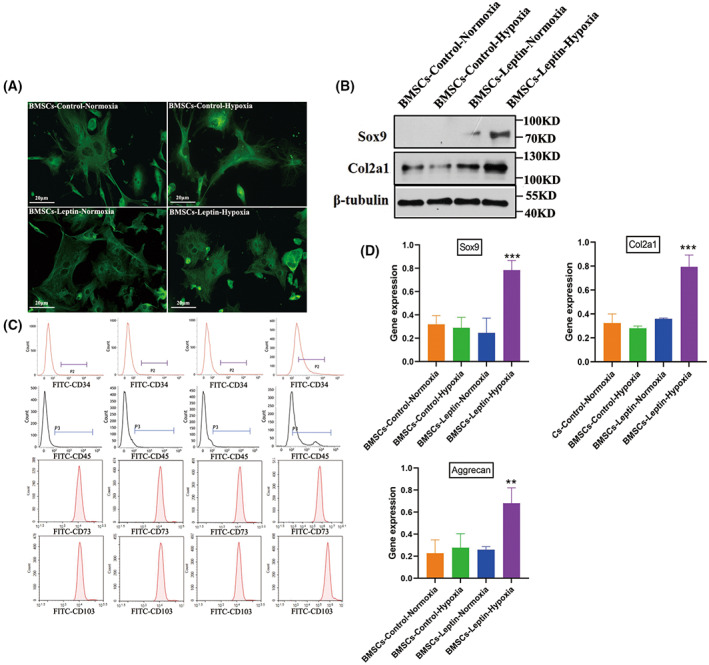
Morphology and characterization of bone marrow mesenchymal stem cells (BMSCs) *in vitro* culture. (A) α‐tubulin stained BMSCs, which were isolated from 8‐week‐old male mice. (B) Western blot analysis of Sox9 and Col2a1 in articular chondrocytes. (C) Flowcytometry analysis of the BMSCs surface markers are CD73 and CD105. (D) Gene expression analysis of Col2a1, aggrecan, and Sox9 in four groups. The expression level in the treatment group was significantly higher than in the degeneration group. Representative microscope images are magnified 400× (scale bar = 20 μm). Error bars indicate standard error (*n* = 3); one‐way ANOVA was used for statistical analysis, **P < 0.01; ***P < 0.001.

In order to clarify the effects and mechanism of leptin on BMSCs differentiation, BMSCs were isolated from the bone marrow of C57BL6/J mice (6 weeks), which were treated with recombinant leptin under normal culture conditions BMSCs‐Lep‐Normoxia(N) and exposed to hypoxia conditions BMSCs‐Lep‐Hypoxia(H) for 48 h *in vitro*. The solvent of leptin, PBS buffer, was used as the control; BMSCs‐Con‐Normoxia(N) and exposed to hypoxia BMSCs‐Con‐Hypoxia(H). Cell morphology was detected: BMSCs showed a typical spindle‐like and elongated fibroblastic shape, while BMSCs‐Lep‐hypoxia volume gradually increased, which together indicated that leptin can stimulate cell morphological changes under hypoxia (Fig. 1A). The BMSCs under normoxia are mostly polygonal cells. BMSCs under hypoxia had abnormal cell morphology, and most cells showed a long spindle shape, and the leptin‐added co‐cultured cells recovered the polygonal shape under hypoxia. The expression profiles of the Sox9, Col2a1 genes were used to assess the different stages of chondrocytes transformation [32]. Western blot showed that the expression of Sox9 was increased in induced BMSCs‐Lep‐H compared to other groups (Fig. 1B). The cell transformation marker phenotype was analyzed by flow cytometry to evaluate the expression of the cultured BMSCs markers, and found that the markers CD73and CD105 were highly expressed, while CD34 and CD45 were of low expression. Under hypoxic conditions, a substantial increase in the cellular differentiation rate was observed in BMSCs‐Lep‐H compared with the BMSCs‐Con‐H. But no significant changes between BMSCs‐con‐N and BMSCs‐Lep‐N under normoxia conditions were observed (Fig. 1C). Gene expression analysis further showed that articular chondrocytes expressed two chondrocytic markers: Sox9 and Col2a1, which BMSCs did not or expressed at very low levels (*P* = 0.0062; Fig. 1D). These findings suggested that BMSCs differentiation into chondrocyte‐like cells could be induced under hypoxia conditions.

### The mitochondrial fusion of BMSCs was increased under the hypoxia condition

**Fig. 2:**
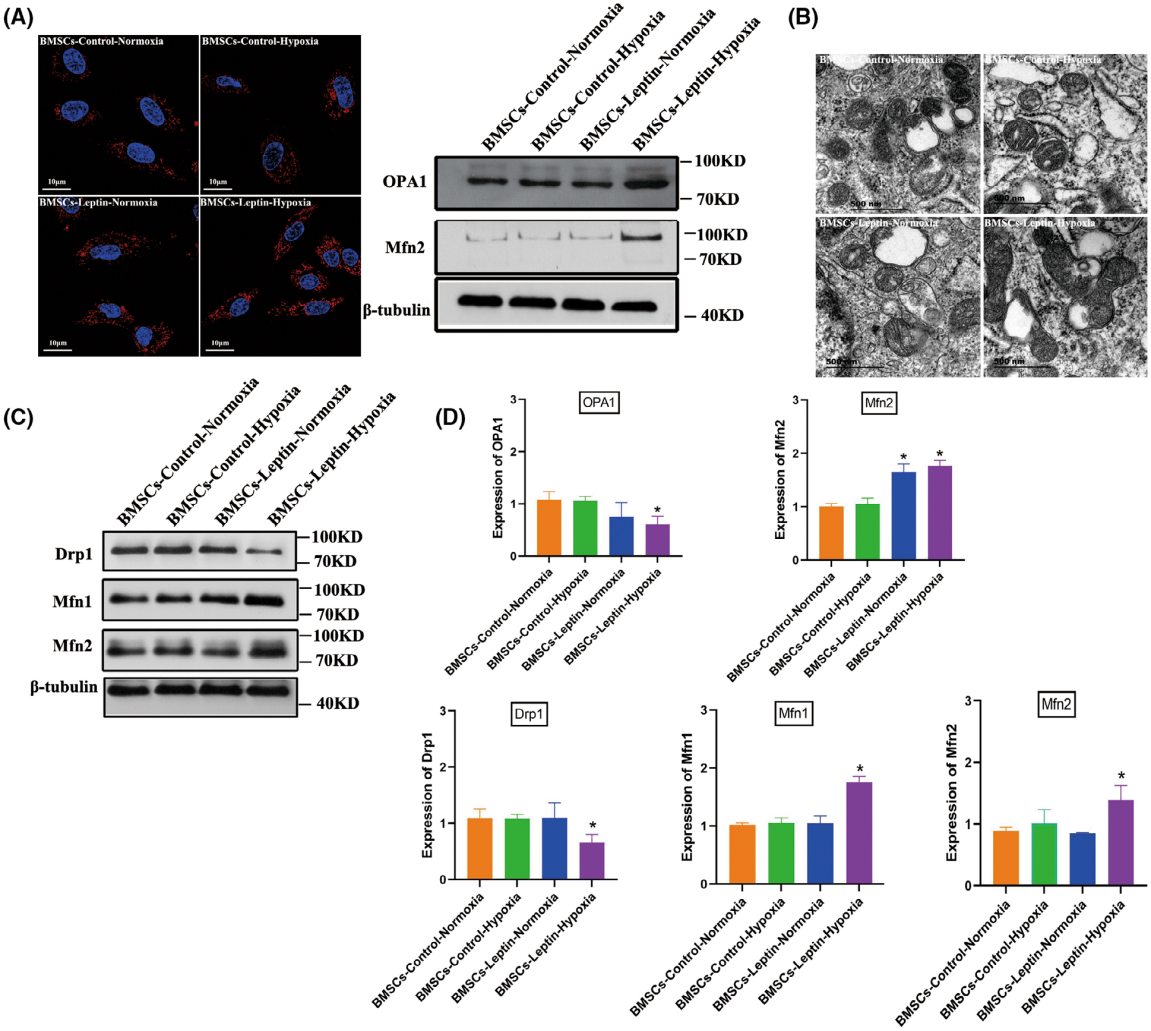
Leptin promotes BMSCs mitochondrial fusion under hypoxia conditions. (A) BMSCs (cocultured with leptin) stained with Mito‐tracker red exhibited an active mitochondrial fusion in hypoxia conditions and the effects of leptin on protein expression of mitochondrial fusion related‐proteins Mfn2, OPA1 under normoxia or hypoxia were clearly quite distinct. (B) Mitochondrial ultrastructures were analyzed by elect micrograph with representative images showing significant changes in mitochondrial length after hypoxic preconditioning (HP) compared with those pretreated with the solvent alone (magnification was set at ×15 000); scale bar, 500 nm. (C) Protein expression implicated mitochondrial homeostasis including fusion and fission were assessed by western blot for BMSCs‐control‐Normoxia (BMSCs cultured under normal oxygen condition) and BMSCs‐control‐hypoxia (BMSCs cultured under hypoxic condition), quantified by densitometry using β‐tubulin as the control. (D) The expression of proteins that control mitochondrial fusion and fission was analyzed, including the mitochondrial fusion protein Mfn1/Mfn2 and mitochondrial fission proteins DRP1. An increase in mitochondrial fusion proteins Mfn1/Mfn2. The expression of mitochondrial fission proteins DRP1 was reduced significantly. Representative microscope images are magnified 600× (scale bar = 10 μm). Error bars indicate standard error (*n* = 3); one‐way ANOVA was used for statistical analysis, *P < 0.05.

Upregulation of mitochondrial metabolism has been linked to mitochondrial fusion in various cells. The mitochondrial morphology change is strongly related to cell transformation [33]. We used the fluorescent probe Mito‐Tracker Red to evaluate mitochondrial fusion. BMSCs‐Con‐N presented sparse and dendritic spines and mitochondria distributed throughout the cells' cytoplasm. Mitochondria of BMSCs‐ Con‐H demonstrated dense and some bold appearance tubular mitochondria (Fig. 2A). In addition, mitochondrial shapes were analyzed and the ultrastructure of mitochondria by TEM (Fig. 2B). Western blot analysis showed that the Drp1 protein was nearly reduced by ∼50% in BMSCs‐Lep‐H. However, the expression of Mfn1 and Mfn2 proteins increased in BMSCs‐Lep‐H (Fig. 2C).

### Mitochondrial regulatory protein PGC‐1α was activated under hypoxia

**Fig. 3:**
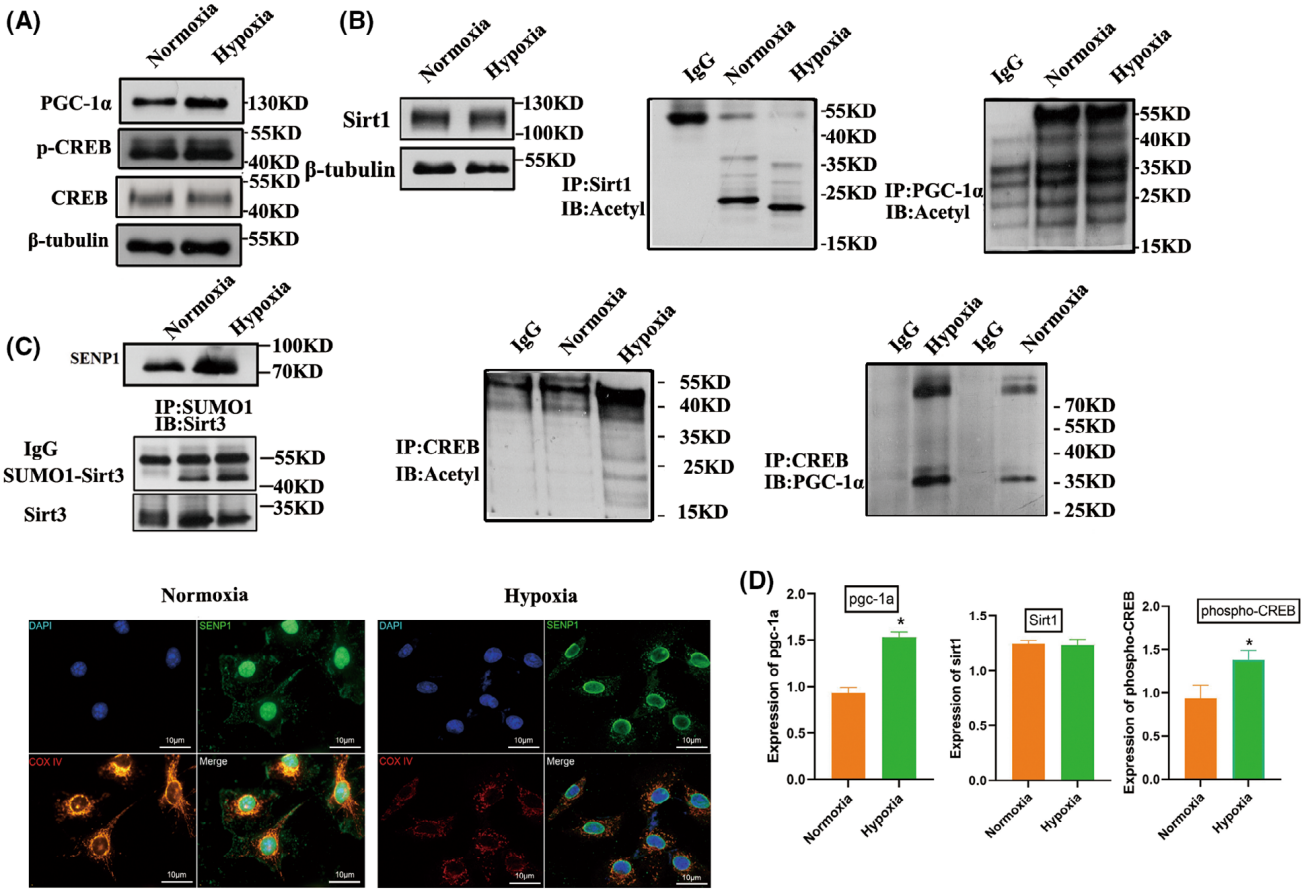
SIRT3 activates the expression of mitochondrial regulatory protein PGC‐1α. (A) Constitutive expression of SIRT3 promotes activation of CREB. Cells were harvested, and phospho‐CREB and total CREB were detected by immunoblot analysis. (B) Effect of SIRT1 acetylation level on PGC‐1α expression level in the hypoxic environment. (C) SENP1 is increased in BMSCs under hypoxia. (D) The differential expression levels identified by western blot using the specific antibody; each experiment was repeated three times. Error bars indicate standard error (*n* = 3), Student's *t*‐test was used for statistical analysis, *P < 0.05.

Having established that mitochondrial fusion increased during the BMSCs differentiation, we tried to seek the mechanism of PGC‐1α activation. We first investigated if the mitochondrial fusions were intimately connected with the increased expression of mitochondrial proteins. In western blot analysis, the twofold increase in PGC‐1α at the protein level was compared to control cells (Fig. 3A). Usually, PGC‐1α is regulated by SIRT1, which activated PGC‐1α through deacetylation [34]. Interestingly, we found no significant change in SIRT1 in transplanted BMSCs, and no significant change in the acetylation level of PGC‐1α (Fig. 3B). We also found that, under hypoxia conditions, the expression of SENP1 was elevated in hypoxic environments, regulatory factor SENP1 transport into the mitochondria, and SENP1 could specifically catalyze the de‐SUMOylation of SIRT3 (Fig. 3C). It has been reported previously that SIRT3 acts on the PGC‐1α promoter [35]. Subsequently, we assessed whether SIRT3 is indispensable for the fusion promoting effects on BMSCs; SIRT3 expression was knocked‐down using siRNA specific for SIRT3, whereas scrambled siRNA (si‐Con) served as the control. The western blot results suggested knockdown of SIRT3 exhibited the opposite results (*P* = 0.0355; Fig. 3D).

### Leptin promotes glycolysis to provide energetic metabolism for transformation

**Fig. 4:**
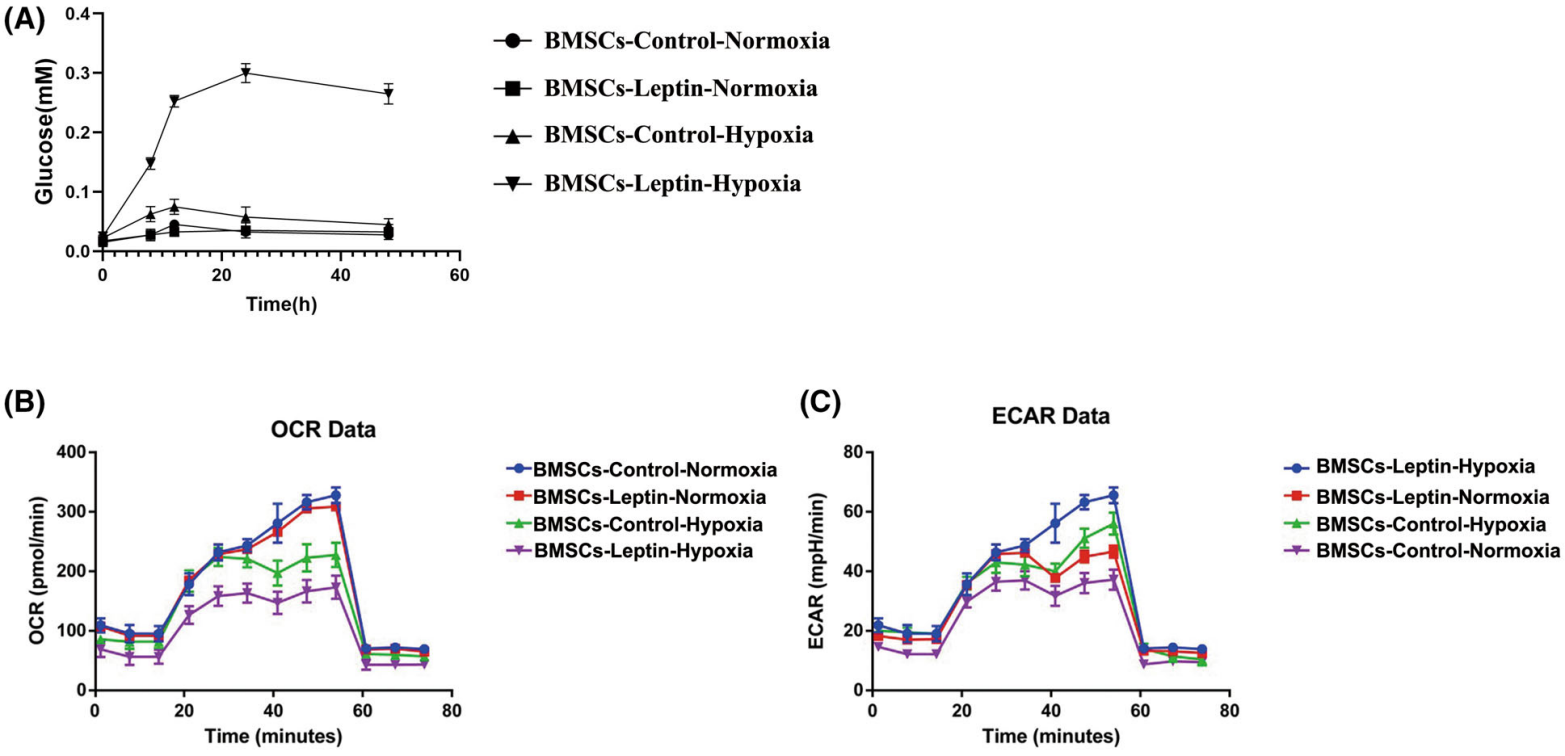
Glycolysis was induced by leptin treatment in the hypoxic environment. (A) Following leptin treatment, BMSCs were exposed to hypoxia and glucose for 48 h. The glucose content was determined using the glucose oxidase method. (B) Mitochondrial oxygen consumption rate (OCR) was determined in BMSCs‐control (without leptin) and BMSCs‐leptin (with leptin) using an Oroboros instrument. (C) Following exposure to hypoxia for 48 h, the rate of extracellular acidification (ECAR) of BMSCs was measured and is shown as a percentage relative to untreated control BMSCs. Error bars indicate standard error (*n* = 3); one‐way ANOVA was used for statistical analysis.

No obvious changes of leptin‐mediated mitochondrial fusion were observed under normal oxygen conditions. We measured the mitochondrial respiratory function, compared with the BMSCs‐Con‐H group, a lower OCR was found in the BMSCs‐Lep‐H groups (Fig. 4A). These data suggested that leptin promoted glycolysis under hypoxia conditions. Glycolysis is key to BMCSs differentiation and energy uptake, especially in the anoxic environment [36, 37]. To determine the changes in glycolysis of BMSCs, the levels of glucose in cells were analyzed by an oxidase method (Fig. 4B). Compared with BMSCs‐con, leptin intervention induced an increase in glucose uptake under hypoxia. In addition, compared with BMSCs‐con‐H, ECAR was increased in BMSCs‐Lep‐H (Fig. 4C), which indicated that leptin could activate glycolysis to generate ATP.

### BMSCs could transform into CEP chondrocytes inhibited intervertebral disc degeneration

**Fig. 5:**
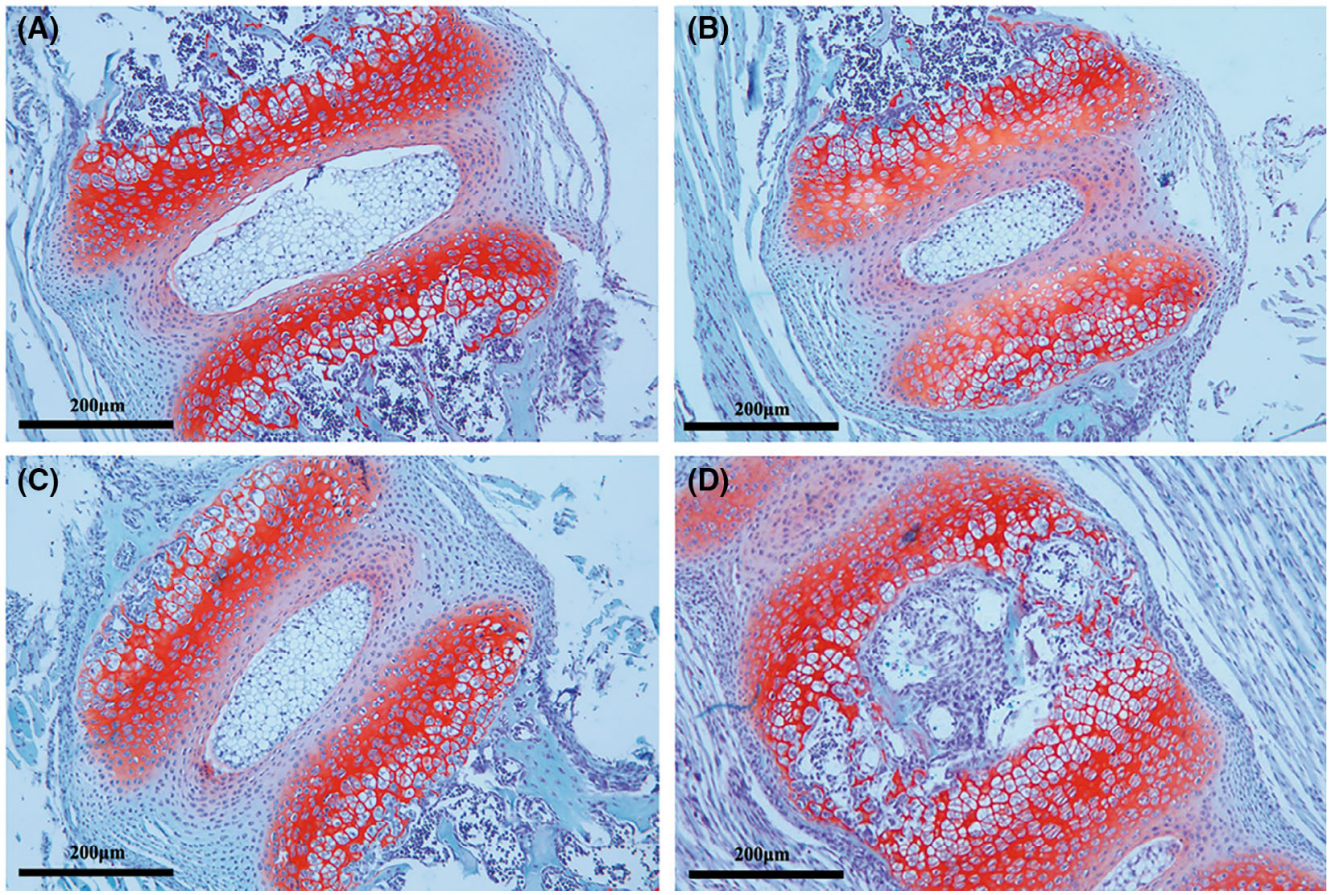
The progressive degeneration of the murine disc was arrested by BMSC. Safranin O staining of intervertebral discs isolated from the normal group. (A) Normal group (B) degeneration group (C) treatment group at 4 weeks. (D) Treatment group at 12 weeks. Representative microscope images are magnified 200× (scale bar = 200 μm).

We used the annular puncture method established in the mouse IVDD models successfully. Subsequent BMSCs were injected into the intervertebral discs 2 weeks later. Samples were uniformly stained with safranin O, and histology observation revealed that the normal CEP chondrocytes were increased and regularly aligned (Fig. 5A). In the degeneration group, the CEP chondrocytes became decreased and disorganized at 4 weeks, and the safranin O staining results were reversed (Fig. 5B). However, such progressive degenerative changes were arrested in the treatment group; the results showed that the cellular components in the cartilage layer were still abundant at 4 weeks postinjection (Fig. 5C). When we measured the 12‐week treatment sample, the structure of the cartilage layer was still relatively intact and the cartilage layer became dominantly positive for safranin O (Fig. 5D).

### The population and expression of chondrocytic markers Col2a1 and Sox9 were increased after BMSCs injection

**Fig. 6:**
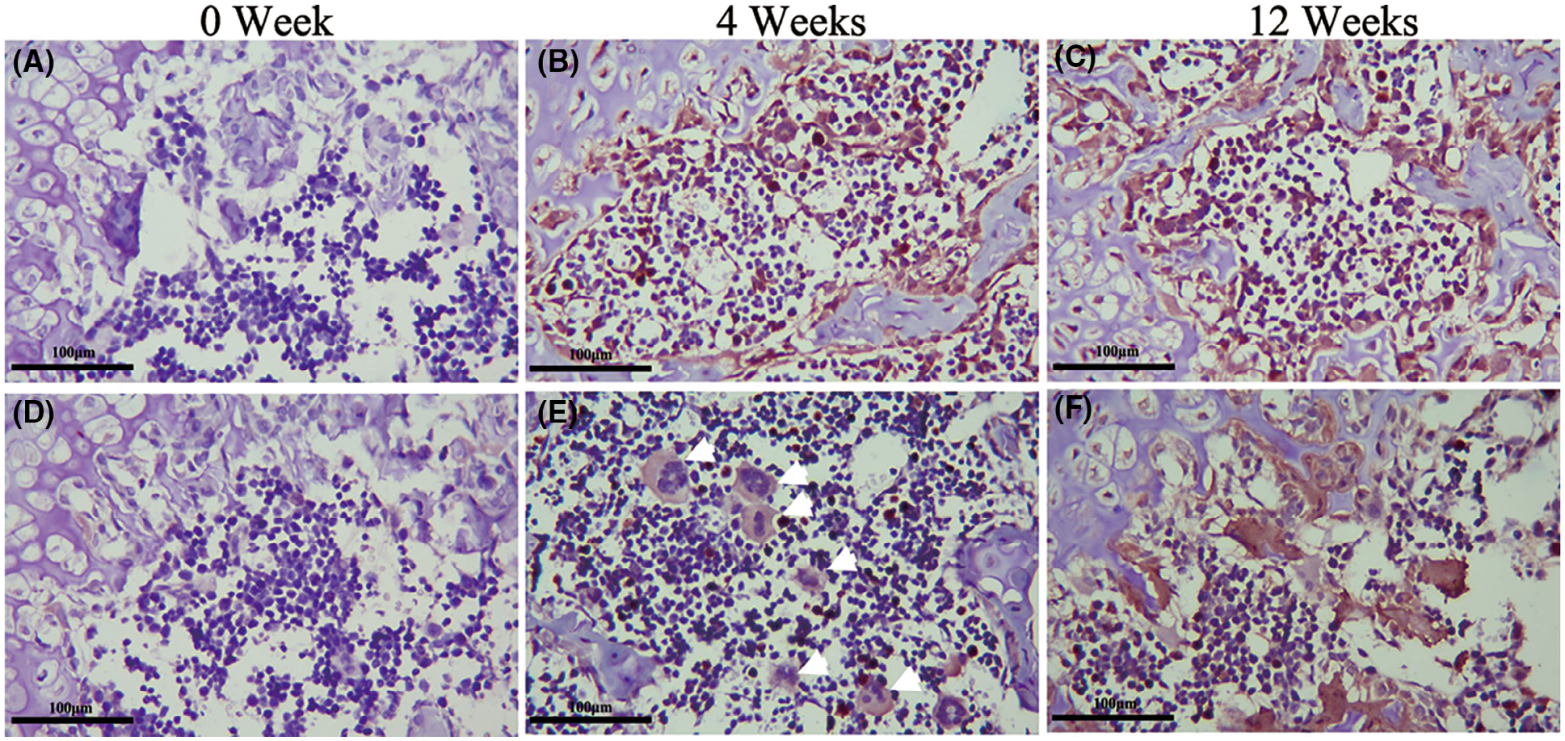
Morphological changes of BMSCs after transplantation. Immunohistochemical (IHC) staining showed that the expression of Sox9 (A–C) and Col2a1 (D–F) at 0/4/12 weeks postinjection. The cartilage markers Sox9 and Col2a1expression level in the treatment group was significantly higher than the degeneration group. Representative microscope images are magnified 200× (scale bar = 100 μm).

Col2a1 and Sox9 are BMSCs positive markers in chondroid differentiation. Intervertebral disc immune staining revealed transplanted BMSCs in cartilage differentiation. No obvious cartilage markers were found in the control group after 0 weeks of BMSCs injection (Sox9 and Col2a1; Fig. 6A,D). Subsequently, Col2a1‐ and Sox9‐positive cells were increased after 4 weeks of treatment (Fig. 6B,E). The Col2a1 and Sox9 protein expression was only moderately increased at 12 weeks (Fig. 6C,F).

### Effects of leptin on chondroid transformation after transplantation of mesenchymal bone marrow stem cells were derived from SIRT3^−/−^ mice

**Fig. 7:**
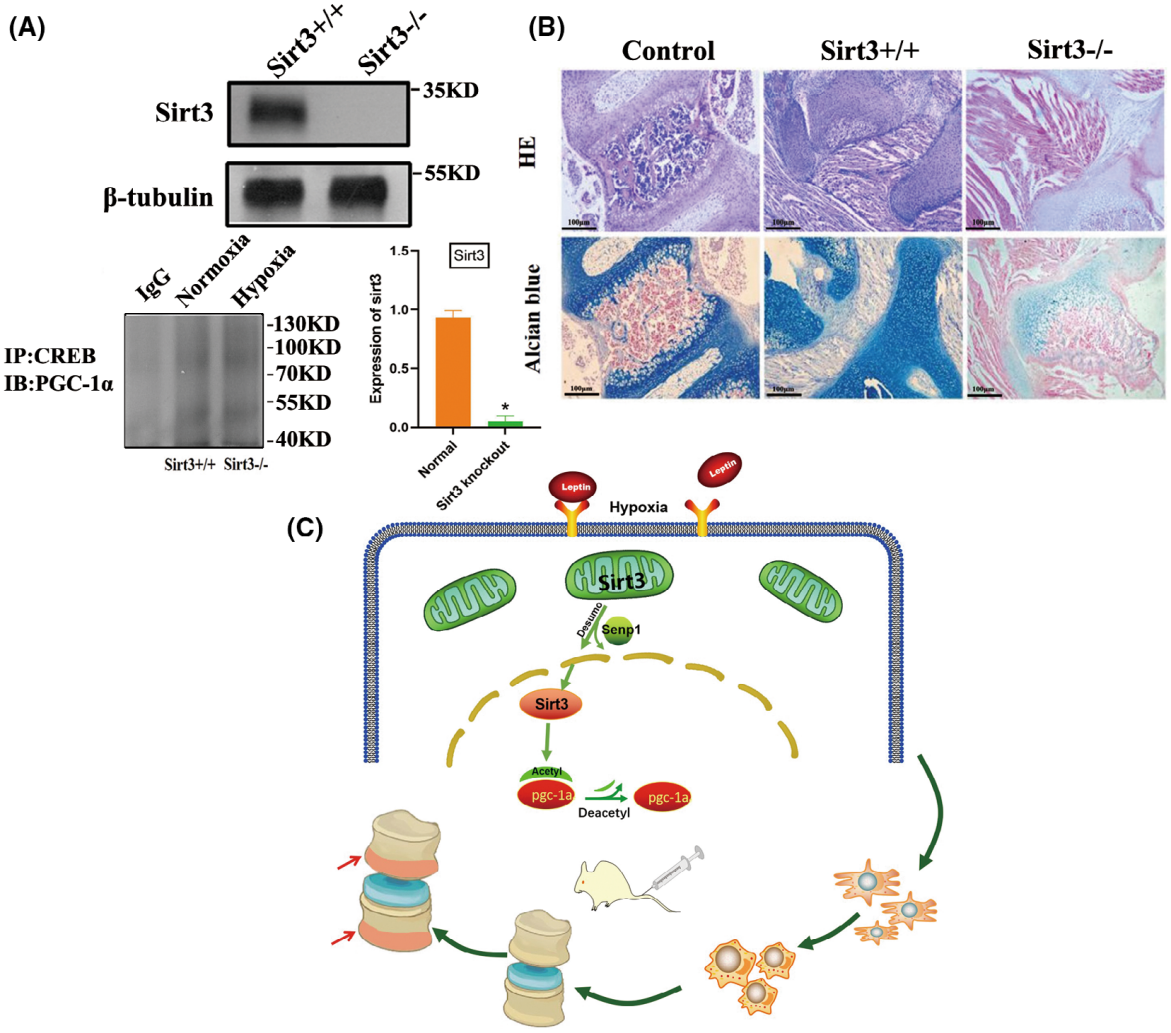
Transplantation BMSCs cells with SIRT3 knockout showed low differentiation. Sirutin 3 (SIRT3) was knocked out in BMSCs of mice (A). The improvement of cartilage level after treatment was observed by Alcian staining. Representative microscope images are magnified 200 × (scale bar = 100 μm) (B). A schematic illustration showed that leptin regulates the ability of mesenchymal stem cells to differentiate into endplate chondrocytes under hypoxic conditions. Mitochondrial fusion promotes the differentiation of BMSCs, and mitochondrial fusion is achieved by SIRT3‐mediated peroxisome proliferator‐activated receptor (PPAR) γ coactivator 1α (PGC‐1α) (C). Error bars indicate standard error (*n* = 6). Student's *t*‐test was used for statistical analysis, *P < 0.05.

To investigate the effect of the SIRT3 gene on the chondroid transformation of BMSCs after intervertebral disc transplantation, we isolated BMSCs from SIRT3^−/−^mouse and SIRT3^+/+^ mouse and transplanted them into the SIRT3^+/+^ mouse circular degeneration model, with seven mice in each group. Western blot was used to assay the knockout efficiency of SIRT3, In the hypoxic environment of BMSCs with SIRT3 knocked out, no obvious activation of CREB into the nucleus was found to bind PGC‐1a (Fig. 7A). Interestingly, Alcian blue staining showed that SIRT3^−/−^ mouse‐derived mesenchymal bone marrow stem cells did not show a significant chondrocyte increase in the transplanted mice (Fig. 7B). The speculative mechanism diagram containing the transformation of BMSCs to vertebral epiphyseal plate chondrocytes regulating activity of SIRT3 through SUMO‐specific protease SENP1 is shown (Fig. 7C). These results suggested that BMSCs lacking the SIRT3 gene were impeded in species differentiation during transplantation.

## Discussion

Bone marrow mesenchymal stem cells (BMSCs) are thought to be able to replace cells lost through aging or damage to tissue cartilage [38, 39]. Its inherent chondrogenic differentiation potential make them an attractive cell population for osteoarticular cartilage repair [40, 41]. To decelerate or stop the degeneration of intervertebral discs it is necessary to understand the homeostasis of chondrogenic differentiation of BMSCs in an anoxic microenvironment. Attenuating disc degeneration though BMSCs transplantation is largely safe and effective based on reported studies [42]. However, the low survival rate limits the therapeutic potential of BMSCs [43, 44]. The enhanced survival rate of transplanted BMSCs may be an effective method to combat disc degeneration caused by cartilage [45]. But improving the survival rate of transplanted cells still faces many challenges. This study attempted to improve the therapeutic efficiency of BMSCs transplantation by promoting the differentiation of BMSCs into chondrocytes. Leptin acts as an adipokine that has a significant regulatory effect on bone metabolism. Previous studies have shown that leptin played an important role in the proliferation and survival of BMSCs. But there were few reports on whether leptin regulates chondrogenic differentiation of bone marrow mesenchymal stem cells. In this study, we confirmed that leptin promotes the differentiation of BMSCs into chondrocytes under hypoxia. The above effects can be regulated by the deacetylase SIRT3 in the SNEP1 signaling pathway. Although BMSCs are pluripotent cells, they have the ability to differentiate osteoblasts, chondrocytes, and adipocytes. We confirmed that in the hypoxic environment, BMSCs tended to be chondrocyte differentiation. Sox9 is a key factor in the differentiation and proliferation of chondrocytes [46]. Sox9 is always actively expressed from chondrocyte progenitors to endstage chondrocytes and is necessary for the maintenance of chondrocyte lineages [45,46]. Type II collagen (encoded by the COL2A1 gene) is a protein marker in hyaluronic cartilage. It is produced and secreted in chondrocytes and plays an important role in the activation of chondrocyte signaling pathways and the maintenance of cartilage matrix functions [47, 48]. This is consistent with previous reports that BMSCs are inhibited in cartilage and osteogenesis in an anoxic environment. However, due to the low differentiation efficiency of BMSCs in the anoxic environment, we revealed that the mitochondrial presentation network of BMSCs treated with leptin (BMSCs‐Lep‐H) were elongated and thickened, suggesting an important role of leptin in promoting mitochondrial fusion under hypoxic conditions. Under normoxia, however, leptin did not increase chondroblast differentiation under normal oxygen conditions. This indicated that leptin maintained the differentiation homeostasis of BMSCs and did not change the expression of chondrogenic marker in BMSCs. Meanwhile, an increase in lactic acid production in BMSCs suggested that the energy required for BMSCs transformation was from leptin‐dependent glycolytic ATP production. In addition, we found that leptin plays an important role in providing energy for cartilage differentiation under hypoxia conditions through glycolysis experiments, and the energy of cell transformation under the hypoxia condition depended on the energy of leptin's glycolysis promotion.

Why did BMSCs show differentiation trends only in the hypoxic environment? SNEP1 acts as a hypoxic response gene and a de‐SUMOylation enzyme [49]; the expression of SNEP1 is increased in a hypoxic environment [50]. In the cell, the activity of SUMOylated SIRT3 deacetylase is inhibited, thus affecting mitochondrial metabolism [51, 52]. We hypothesized that the phenomenon was linked to the differential regulation of SNEP1, SNEP1 deSUMOylates, and SIRT3 activation. Interestingly, how does the mitochondrial localization of SIRT3 activate nuclear genes such as PGC‐1α. It was demonstrated previously that phosphorylated CREB binding to the PGC‐1α promoter stimulated the expression of PGC‐1α, and the CREB phosphorylation was regulated by SIRT3. The SIRT1 signaling pathway did not change significantly. The changes of SIRT1 in PGC‐1α tested by us did not show significant changes. The SIRT3/PGC‐1a pathway is an important regulatory protein of mitochondria, and also a key regulatory pathway of chondrocyte terminal differentiation, which is necessary to maintain the homeostasis of chondrogenic differentiation.

*In vivo*, transgenic mouse models may further help us understand the mechanisms of IVDD regeneration based on BMSCs. Our data suggested that the high expression of Sox9 in BMSCs‐treated intervertebral discs indicated that the BMSCs differentiate into chondropheno types. The increase in Col2a1 from 4 to 24 weeks suggested that BMSC‐treated intervertebral discs became a chondrocyte. Leptin plays an active role in promoting BMSCs transformation to chondrocytes, which is associated with mitochondrial fusion. Furthermore, transformation of BMSCs derived from SIRT3^−/−^ mice was blocked during transplantation, which further proved that SIRT3 played a key regulatory role in the transplantation and differentiation of BMSCs. At the same time, the transplanted BMSCs successful transformation to chondrogenic lineages under leptin induction after 4 weeks later, and the intervertebral disc cartilage layer was increased.

Our findings of leptin's role in the process of regeneration based on BMSCs transformation, providing insights in the design of new regenerative therapies for IVDD. But there were also some limitations in this study. First of all, although we found that BMSCs softening under hypoxia showed a chondrogenic differentiation trend, its mechanism has not been further investigated. Second, p‐CREB is indeed a marker of CREB activation, but it further verifies how PGC‐1α is regulated by a nuclear entry. In the future, we will conduct more carefully designed studies on the upstream and downstream genes of the SENP1/SIRT3/PGC‐1α pathway to further explore the regulatory role of leptin in the BMSCs signaling pathway.

## Conclusion

Here we demonstrated, first that BMSCs transplantation in anoxic environments is improved by leptin‐mediated mitochondrial fusion, which promotes cells transformation. Differentiation of BMSCs depends on SIRT3 activation via the SENP1‐mediated deSUMOylation, which translocate into the nucleus after CREB phosphorylation and upregulation of PGC‐1α. Leptin can provide glucose and ATP for mitochondria fusion by affecting glycolysis. This may provide a new pharmacological approach on IDD diseases.

## Conflict of interest

The authors declare no conflicts of interest.

## Author contributions

XL conceived the project, analyzed the data, and wrote the article. XF and HL helped to design the study, performed the experiments, participated in analyzing the dataset, and writing the article. YG and WW assisted with sample collection and data analysis, and revised the article. ZL and YS reviewed the article and provided substantial advice through experimental work. All authors read though the article and approved the final version.

## Data Availability

The data are available from the corresponding author on reasonable request.

## References

[1] Chou D , Samartzis D , Bellabarba C , Patel A , Luk KD , Kisser JM , et al. Degenerative magnetic resonance imaging changes in patients with chronic low back pain: a systematic review. Spine (Phila Pa 1976). 2011;36:S43–53. 21952189 10.1097/BRS.0b013e31822ef700

[2] Livshits G , Popham M , Malkin I , Sambrook PN , Macgregor AJ , Spector T , et al. Lumbar disc degeneration and genetic factors are the main risk factors for low back pain in women: the UK twin spine study. Ann Rheum Dis. 2011;70:1740–5. 21646416 10.1136/ard.2010.137836 PMC3171106

[3] Fields AJ , Han M , Krug R , Lotz JC . Cartilaginous end plates: quantitative MR imaging with very short echo times‐orientation dependence and correlation with biochemical composition. Radiology. 2015;274:482–9. 25302832 10.1148/radiol.14141082 PMC4314292

[4] Nombela‐Arrieta C , Ritz J , Silberstein LE . The elusive nature and function of mesenchymal stem cells. Nat Rev Mol Cell Biol. 2011;12:126–31. 21253000 10.1038/nrm3049 PMC3346289

[5] Saeed H , Ahsan M , Saleem Z , Iqtedar M , Islam M , Danish Z , et al. Mesenchymal stem cells (MSCs) as skeletal therapeutics – an update. J Biomed Sci. 2016;23:41. 27084089 10.1186/s12929-016-0254-3 PMC4833928

[6] Bach FC , Willems N , Penning LC , Ito K , Meij BP , Tryfonidou MA . Potential regenerative treatment strategies for intervertebral disc degeneration in dogs. BMC Vet Res. 2014;10:3. 24387033 10.1186/1746-6148-10-3 PMC3914844

[7] Peroglio M , Douma LS , Caprez TS , Janki M , Benneker LM , Alini M , et al. Intervertebral disc response to stem cell treatment is conditioned by disc state and cell carrier: an ex vivo study. J Orthop Translat. 2017;9:43–51. 29662798 10.1016/j.jot.2017.03.003 PMC5822953

[8] Bianco P , Riminucci M , Gronthos S , Robey PG . Bone marrow stromal stem cells: nature, biology, and potential applications. Stem Cells. 2001;19:180–92. 11359943 10.1634/stemcells.19-3-180

[9] Guo J , Wang R , Liu D . Bone marrow‐derived mesenchymal stem cells ameliorate sepsis‐induced acute kidney injury by promoting mitophagy of renal tubular epithelial cells via the SIRT1/parkin Axis. Front Endocrinol (Lausanne). 2021;12:639165. 34248837 10.3389/fendo.2021.639165 PMC8267935

[10] Zhu B , Xue F , Zhang C , Li G . LMCD1 promotes osteogenic differentiation of human bone marrow stem cells by regulating BMP signaling. Cell Death Dis. 2019;10:647. 31501411 10.1038/s41419-019-1876-7 PMC6733937

[11] Havel PJ , Kasim‐Karakas S , Mueller W , Johnson PR , Gingerich RL , Stern JS . Relationship of plasma leptin to plasma insulin and adiposity in normal weight and overweight women: effects of dietary fat content and sustained weight loss. J Clin Endocrinol Metab. 1996;81:4406–13. 8954050 10.1210/jcem.81.12.8954050

[12] Zhang Y , Proenca R , Maffei M , Barone M , Leopold L , Friedman JM . Positional cloning of the mouse obese gene and its human homologue. Nature. 1994;372:425–32. 7984236 10.1038/372425a0

[13] Tartaglia LA . The leptin receptor. J Biol Chem. 1997;272:6093–6. 9102398 10.1074/jbc.272.10.6093

[14] Barrichon M , Hadi T , Wendremaire M , Ptasinski C , Seigneuric R , Marcion G , et al. Dose‐dependent biphasic leptin‐induced proliferation is caused by non‐specific IL‐6/NF‐kappaB pathway activation in human myometrial cells. Br J Pharmacol. 2015;172:2974–90. 25653112 10.1111/bph.13100 PMC4459017

[15] Guo Z , Jiang H , Xu X , Duan W , Mattson MP . Leptin‐mediated cell survival signaling in hippocampal neurons mediated by JAK STAT3 and mitochondrial stabilization. J Biol Chem. 2008;283:1754–63. 17993459 10.1074/jbc.M703753200

[16] Hehar H , Ma I , Mychasiuk R . Intergenerational transmission of paternal epigenetic marks: mechanisms influencing susceptibility to post‐concussion symptomology in a rodent model. Sci Rep. 2017;7:7171. 28769086 10.1038/s41598-017-07784-7 PMC5541091

[17] Scheller EL , Song J , Dishowitz MI , Soki FN , Hankenson KD , Krebsbach PH . Leptin functions peripherally to regulate differentiation of mesenchymal progenitor cells. Stem Cells. 2010;28:1071–80. 20506495 10.1002/stem.432 PMC2907517

[18] Douros JD , Baltzegar DA , Reading BJ , Seale AP , Lerner DT , Grau EG , et al. Leptin stimulates cellular glycolysis through a STAT3 dependent mechanism in tilapia. Front Endocrinol (Lausanne). 2018;9:465. 30186233 10.3389/fendo.2018.00465 PMC6110908

[19] Toda C , Shiuchi T , Kageyama H , Okamoto S , Coutinho EA , Sato T , et al. Extracellular signal‐regulated kinase in the ventromedial hypothalamus mediates leptin‐induced glucose uptake in red‐type skeletal muscle. Diabetes. 2013;62:2295–307. 23530005 10.2337/db12-1629 PMC3712028

[20] Burmistrova O , Olias‐Arjona A , Lapresa R , Jimenez‐Blasco D , Eremeeva T , Shishov D , et al. Targeting PFKFB3 alleviates cerebral ischemia‐reperfusion injury in mice. Sci Rep. 2019;9:11670. 31406177 10.1038/s41598-019-48196-z PMC6691133

[21] Palikaras K , Lionaki E , Tavernarakis N . Balancing mitochondrial biogenesis and mitophagy to maintain energy metabolism homeostasis. Cell Death Differ. 2015;22:1399–401. 26256515 10.1038/cdd.2015.86 PMC4532782

[22] Lin J , Handschin C , Spiegelman BM . Metabolic control through the PGC‐1 family of transcription coactivators. Cell Metab. 2005;1:361–70. 16054085 10.1016/j.cmet.2005.05.004

[23] Puigserver P , Rhee J , Lin J , Wu Z , Yoon JC , Zhang CY , et al. Cytokine stimulation of energy expenditure through p38 MAP kinase activation of PPARgamma coactivator‐1. Mol Cell. 2001;8:971–82. 11741533 10.1016/s1097-2765(01)00390-2

[24] Li L , Pan R , Li R , Niemann B , Aurich AC , Chen Y , et al. Mitochondrial biogenesis and peroxisome proliferator‐activated receptor‐γ coactivator‐1α (PGC‐1α) deacetylation by physical activity: intact adipocytokine signaling is required. Diabetes. 2011;60:157–67. 20929977 10.2337/db10-0331 PMC3012167

[25] Wang H , Peiris TH , Mowery A , Le Lay J , Gao Y , Greenbaum LE . CCAAT/enhancer binding protein‐beta is a transcriptional regulator of peroxisome‐proliferator‐activated receptor‐gamma coactivator‐1alpha in the regenerating liver. Mol Endocrinol. 2008;22:1596–605. 18467525 10.1210/me.2007-0388 PMC2453599

[26] Gerhart‐Hines Z , Rodgers JT , Bare O , Lerin C , Kim SH , Mostoslavsky R , et al. Metabolic control of muscle mitochondrial function and fatty acid oxidation through SIRT1/PGC‐1alpha. EMBO J. 2007;26:1913–23. 17347648 10.1038/sj.emboj.7601633 PMC1847661

[27] Hirschey MD . Old enzymes, new tricks: SIRTuins are NAD(+)‐dependent de‐acylases. Cell Metab. 2011;14:718–9. 22100408 10.1016/j.cmet.2011.10.006 PMC3830953

[28] Aquilano K , Vigilanza P , Baldelli S , Pagliei B , Rotilio G , Ciriolo MR . Peroxisome proliferator‐activated receptor gamma co‐activator 1alpha (PGC‐1alpha) and SIRTuin 1 (SIRT1) reside in mitochondria: possible direct function in mitochondrial biogenesis. J Biol Chem. 2010;285:21590–9. 20448046 10.1074/jbc.M109.070169 PMC2898414

[29] Nemoto S , Fergusson MM , Finkel T . SIRT1 functionally interacts with the metabolic regulator and transcriptional coactivator PGC‐1{alpha}. J Biol Chem. 2005;280:16456–60. 15716268 10.1074/jbc.M501485200

[30] Ryu HY , Zhao D , Li J , Su D , Hochstrasser M . Histone sumoylation promotes Set3 histone‐deacetylase complex‐mediated transcriptional regulation. Nucleic Acids Res. 2020;48:12151–68. 33231641 10.1093/nar/gkaa1093 PMC7708062

[31] Schwer B , North BJ , Frye RA , Ott M , Verdin E . The human silent information regulator (sir)2 homologue hSIRT3 is a mitochondrial nicotinamide adenine dinucleotide‐dependent deacetylase. J Cell Biol. 2002;158:647–57. 12186850 10.1083/jcb.200205057 PMC2174009

[32] Bhattacharjee M , Coburn J , Centola M , Murab S , Barbero A , Kaplan DL , et al. Tissue engineering strategies to study cartilage development, degeneration and regeneration. Adv Drug Deliv Rev. 2015;84:107–22. 25174307 10.1016/j.addr.2014.08.010

[33] Wai T , Langer T . Mitochondrial dynamics and metabolic regulation. Trends Endocrinol Metab. 2016;27:105–17. 26754340 10.1016/j.tem.2015.12.001

[34] Roy Chowdhury SK , Smith DR , Saleh A , Schapansky J , Marquez A , Gomes S , et al. Impaired adenosine monophosphate-activated protein kinase signalling in dorsal root ganglia neurons is linked to mitochondrial dysfunction and peripheral neuropathy in diabetes. Brain. 2012;135:1751–66. 22561641 10.1093/brain/aws097 PMC3359752

[35] Sin J , Andres AM , Taylor DJ , Weston T , Hiraumi Y , Stotland A , et al. Mitophagy is required for mitochondrial biogenesis and myogenic differentiation of C2C12 myoblasts. Autophagy. 2016;12:369–80. 26566717 10.1080/15548627.2015.1115172 PMC4836019

[36] Latzko L , Schopf B , Weissensteiner H , Fazzini F , Fendt L , Steiner E , et al. Implications of standardized uptake values of Oral squamous cell carcinoma in PET‐CT on prognosis, tumor characteristics and mitochondrial DNA Heteroplasmy. Cancers (Basel). 2021;13:2273. 34068489 10.3390/cancers13092273 PMC8125984

[37] Manevski M , Muthumalage T , Devadoss D , Sundar IK , Wang Q , Singh KP , et al. Cellular stress responses and dysfunctional mitochondrial‐cellular senescence, and therapeutics in chronic respiratory diseases. Redox Biol. 2020;33:101443. 32037306 10.1016/j.redox.2020.101443 PMC7251248

[38] Lu CH , Yeh TS , Yeh CL , Fang YH , Sung LY , Lin SY , et al. Regenerating cartilages by engineered ASCs: prolonged TGF‐beta3/BMP‐6 expression improved articular cartilage formation and restored zonal structure. Mol Ther. 2014;22:186–95. 23851345 10.1038/mt.2013.165 PMC3978803

[39] Shirbaghaee Z , Hassani M , Heidari Keshel S , Soleimani M . Emerging roles of mesenchymal stem cell therapy in patients with critical limb ischemia. Stem Cell Res Ther. 2022;13:462. 36068595 10.1186/s13287-022-03148-9 PMC9449296

[40] Diederichs S , Shine KM , Tuan RS . The promise and challenges of stem cell‐based therapies for skeletal diseases: stem cell applications in skeletal medicine: potential, cell sources and characteristics, and challenges of clinical translation. Bioessays. 2013;35:220–30. 22948900 10.1002/bies.201200068 PMC4891940

[41] Khurana A , Chapelin F , Beck G , Lenkov OD , Donig J , Nejadnik H , et al. Iron administration before stem cell harvest enables MR imaging tracking after transplantation. Radiology. 2013;269:186–97. 23850832 10.1148/radiol.13130858 PMC3781357

[42] Kim H , Lee MJ , Bae EH , Ryu JS , Kaur G , Kim HJ , et al. Comprehensive molecular profiles of functionally effective MSC‐derived extracellular vesicles in immunomodulation. Mol Ther. 2020;28:1628–44. 32380062 10.1016/j.ymthe.2020.04.020 PMC7335740

[43] Liu H , Liu S , Qiu X , Yang X , Bao L , Pu F , et al. Donor MSCs release apoptotic bodies to improve myocardial infarction via autophagy regulation in recipient cells. Autophagy. 2020;16:2140–55. 31959090 10.1080/15548627.2020.1717128 PMC7751634

[44] Muller‐Ehmsen J , Krausgrill B , Burst V , Schenk K , Neisen UC , Fries JW , et al. Effective engraftment but poor mid‐term persistence of mononuclear and mesenchymal bone marrow cells in acute and chronic rat myocardial infarction. J Mol Cell Cardiol. 2006;41:876–84. 16973174 10.1016/j.yjmcc.2006.07.023

[45] Yang H , Cao C , Wu C , Yuan C , Gu Q , Shi Q , et al. TGF‐betal suppresses inflammation in cell therapy for intervertebral disc degeneration. Sci Rep. 2015;5:13254. 26289964 10.1038/srep13254 PMC4542522

[46] Liang B , Mamidi MK , Samsa WE , Chen Y , Lee B , Zheng Q , et al. Targeted and sustained Sox9 expression in mouse hypertrophic chondrocytes causes severe and spontaneous osteoarthritis by perturbing cartilage homeostasis. Am J Transl Res. 2020;12:1056–69. 32269734 PMC7137053

[47] Hou M , Zhang Y , Zhou X , Liu T , Yang H , Chen X , et al. Kartogenin prevents cartilage degradation and alleviates osteoarthritis progression in mice via the miR‐146a/NRF2 axis. Cell Death Dis. 2021;12:483. 33986262 10.1038/s41419-021-03765-x PMC8119954

[48] Jeevithan E , Bao B , Bu Y , Zhou Y , Zhao Q , Wu W . Type II collagen and gelatin from silvertip shark (*Carcharhinus albimarginatus*) cartilage: isolation, purification, physicochemical and antioxidant properties. Mar Drugs. 2014;12:3852–73. 24979271 10.3390/md12073852 PMC4113802

[49] Sun XX , Chen Y , Su Y , Wang X , Chauhan KM , Liang J , et al. SUMO protease SENP1 deSUMOylates and stabilizes c‐Myc. Proc Natl Acad Sci USA. 2018;115:10983–8. 30305424 10.1073/pnas.1802932115 PMC6205424

[50] Yan Z , Cheng M , Hu G , Wang Y , Zeng S , Huang A , et al. Positive feedback of SuFu negating protein 1 on hedgehog signaling promotes colorectal tumor growth. Cell Death Dis. 2021;12:199. 33608498 10.1038/s41419-021-03487-0 PMC7896051

[51] Hsu HL , Liao PL , Cheng YW , Huang SH , Wu CH , Li CH , et al. Chloramphenicol induces autophagy and inhibits the hypoxia inducible Factor‐1 alpha pathway in non‐small cell lung cancer cells. Int J Mol Sci. 2019;20:157. 30609861 10.3390/ijms20010157 PMC6337541

[52] Wang T , Cao Y , Zheng Q , Tu J , Zhou W , He J , et al. SENP1‐SIRT3 signaling controls mitochondrial protein acetylation and metabolism. Mol Cell. 2019;75:823–34.e5. 31302001 10.1016/j.molcel.2019.06.008

